# Fractionation, rearrangement and subgenome dominance

**DOI:** 10.1093/bioinformatics/bts392

**Published:** 2012-09-03

**Authors:** David Sankoff, Chunfang Zheng

**Affiliations:** Department of Mathematics and Statistics, University of Ottawa, Ottawa, Canada K1N 6N5

## Abstract

**Motivation:** Fractionation is arguably the greatest cause of gene order disruption following whole genome duplication, causing severe biases in chromosome rearrangement-based estimates of evolutionary divergence.

**Results:** We show how to correct for this bias almost entirely by means of a ‘consolidation’ algorithm for detecting and suitably transforming identifiable regions of fractionation. We characterize the process of fractionation and the performance of the algorithm through realistic simulations. We apply our method to a number of core eudicot genomes, we and by studying the fractionation regions detected, are able to address topical issues in polyploid evolution.

**Availability and implementation:** Code for the consolidation algorithm, and sample data, is available at: http://137.122.149.195/Software/Fractionation/fractionation.html

**Contact:** sankoff@uottawa.ca

## 1 INTRODUCTION

Fractionation (Langham *et al*., 2004), the loss of duplicate genes after whole genome duplication (WGD), causes more gene order disruption than classical chromosomal rearrangements such as inversion or reciprocal translocation. WGD and fractionation are particularly prevalent in flowering plants (Soltis *et al*., 2009), where the cycle of the two processes also involves the constant excision of excess non-coding DNA (Eckard, 2001; Freeling *et al*., 2012), a major difference between these organisms and other evolutionary domains, such as the mammals.

Gene order disruption follows from the partly random choice of which of the two copies is deleted, i.e. which of two homeologous chromosomes retains the remaining single copy of the gene. This process was first hypothesized by Wolfe and Shields in their 1997 demonstration of the ancient WGD of *Saccharomyces cerevisiae* (Wolfe and Shields, 1997), suggesting ‘… this is the result of random deletion of individual duplicated genes from one or other chromosome subsequent to the initial duplication of the whole region’. This loss pattern was further highlighted years later by the comparison of the *S. cerevisiae* gene order with that of related diploid yeasts by Dietrich *et al*. (2004) and Kellis *et al*. (2004), who called it ‘interleaving’, while Freeling was coining the usage ‘fractionation’ in the context of plant genomics.

**Fig. 1.**
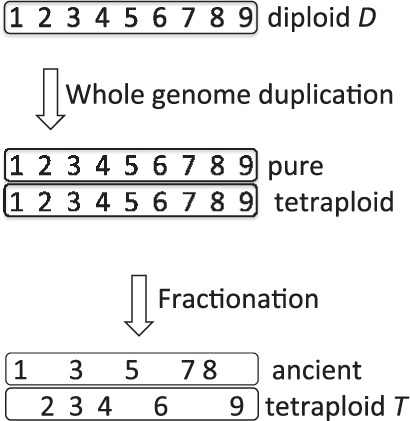
Fractionation leading to different adjacencies in diploid and ancient tetraploid

When a number of adjacent duplicate pairs lose a group of their redundant genes from one homeolog and another group from the other homeolog, as in Figure 1, methods for inferring the rearrangement distances between the WGD descendant *T* (referred to as an ‘ancient tetraploid’ despite being a present-day genome, long since re-diploidized) and an unduplicated sister genome *D* automatically infer that there are rearrangement breakpoints where adjacency no longer exists between the two groups of single-copy survivors. This artificially inflates the inferred number of reciprocal translocations and greatly exaggerates the overall amount of chromosomal rearrangement between the two sister genomes. The first goal of our work is to be able to computationally detect, characterize and correct for this impediment to the study of evolution.

Key to our method is the identification and isolation of ‘fractionation intervals’, regions in both the ancient tetraploid and its sister diploid that have become entirely single copy and may or may not have been rearranged internally but have (so far) been unaffected by rearrangements exchanging genes from within the interval and genes external to the interval. The second goal of our work is to inventory these regions across the two genomes to that they can be studied quantitatively. The statistical properties of the intervals bear on current topics of interest in plant evolutionary genomics, whether duplicated genes are silenced or deleted one by one or through the deletion of longer stretches of DNA, whether a fractionation regions tends to lose genes largely from one of the homeologous chromosomal segments or equally from the two and on the question of subgenome dominance, i.e. whether any such bias toward one homeolog persists from the original WGD event and is unaffected by chromosome shuffling.

## 2 METHODS

### 2.1 Methodological preamble

In this article, we focus on the evolutionary divergence of a genome after WGD compared with a related diploid. The mathematics of genome comparison at the level of gene order or conserved synteny blocks is well worked out [reviewed in (Fertin *et al*., 1974)] when the two genomes have identical complements of single-copy genes only, but the problem is more difficult when they have different gene complements or genes that occur two or more times in a genome. There are many approaches to the comparison of genomes with duplicates, as reviewed in (El-Mabrouk and Sankoff, 2012). These range from ‘guided genome halving’ where single-copy genes may simply be excluded from ancient tetraploid (Zheng *et al*., 2008) or included in an *ad hoc* way (Sankoff *et al*., 2009), through ‘duplication-loss’ models where the ordering of loss and duplication events is the focus (Marron *et al*., 2004), to the ‘exemplar method’ where it is the duplicate genes that are excluded or rather reduced to single-copy status in an optimal way before the comparison and everything in between (Sankoff, 1999). Although all of these are well motivated in particular contexts, they all invoke objective functions that are largely irrelevant to fractionation.

The central datum in genome comparison algorithms is generally the ‘gene adjacency’. Ideally, the genes are oriented, i.e. their 5’- and 3’-ends are distinguished, and when we speak of two genes being adjacent, we mean that the 3’-end of one is adjacent to the 5’-end of the next one, if they are on the same DNA strand or that their two 5’-ends (or two 3’-ends) are adjacent if they are on complementary strands. An adjacency is thus defined by two specific gene ends and not by two genes.

Decomposing a genome into its adjacencies, to take advantage of genome comparison algorithms, loses no information in the case of diploid genomes if all genes are present in only one copy, since then the genome is completely determined by its adjacencies. This equivalence between genomes and sets of adjacencies may break down when duplicate genes occur in large numbers. Nevertheless, in this article, we will use the adjacencies directly to bypass the arbitrary use of objective functions and other assumptions inherent in most comparison algorithms. In particular, it will be appropriate in the WGD-fractionation context to simply compare the sets of adjacencies that occur at least once in a genome as the relevant representation of that genome.

### 2.2 Excess adjacencies as a measure of rearrangement

As a doubled genome evolves through genome rearrangement and/or fractionation, there is a direct effect on the inventory of oriented gene adjacencies. Initially, a new tetraploid and an unduplicated sister genome will have the exact same adjacencies, although these will have multiplicity 2 in the tetraploid. Over time, rearrangement will change the adjacencies in the diploid, without appreciably changing the total number of adjacencies. (This number can change only as a result of the rare events of chromosomal fission or fusion, whereas it does not change after the more frequent events of inversion or reciprocal translocation.) At the same time, in the tetraploid, both rearrangement and fractionation will add new adjacencies without necessarily losing the original ones, since an evolutionary event need only affect one gene copy or one homeolog at a time, leaving the other intact. Thus, the total number of different adjacencies in the diploid and the ancient tetraploid *T*, in excess of the number in the diploid *D* alone, measures the degree of evolutionary divergence.

**Fig. 2.**
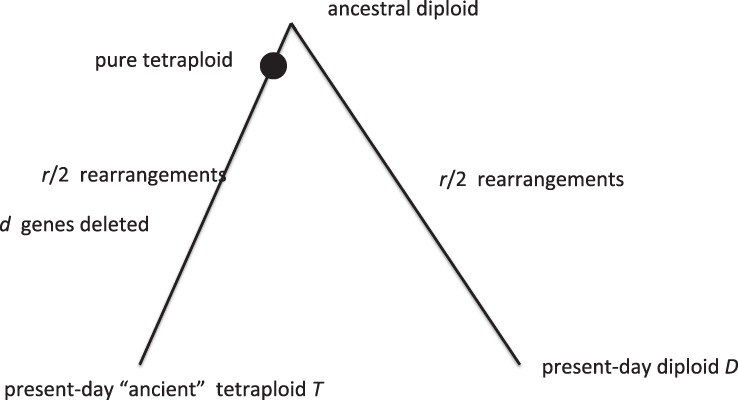
Schema for simulation of divergence between an ancient tetraploid and a sister diploid

We carried out simulations of the schema in Figure 2 to quantify the effect of rearrangement on the number of adjacencies, involving random chromosomal inversions and reciprocal translocations in the proportions 10:1. The simulations modeled a diploid with 24 000 genes randomly divided among 20 chromosomes. The two breakpoints of each rearrangement were chosen at random on the same chromosome for inversions, and on two randomly chosen chromosomes, for translocations. A separate series of simulations modeled the inversions as short (random inversion length drawn from a geometric distribution, with mean length of five genes) and unrestricted, with two random end points, in the proportion 20:1. The differences in the results between the two kinds of model were negligible in all analyses in this article, including the one in Section 4.4 later.

All simulations were run 20 times, and their average values were plotted. Because of the large number of genes in the simulations, variability among the replicates would be almost imperceptible on the scale of the diagrams to be presented here.

**Fig. 3.**
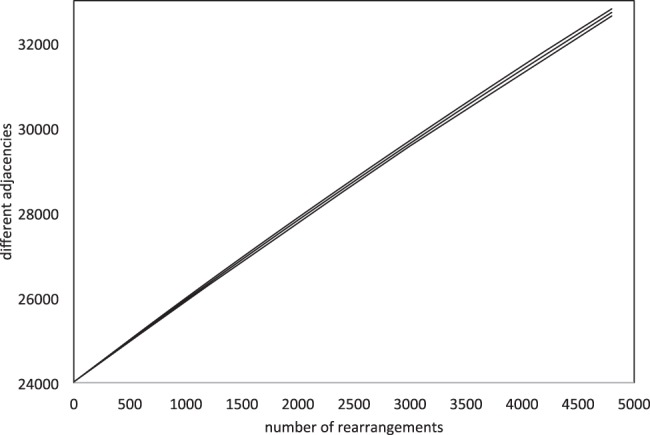
Increase of excess adjacencies in simulations as a function of number of rearrangements *r*. Vertical axis represents total number of different adjacencies in *D* and *T* combined. Showing curves for mean and +/− two SD

Figure 3 shows the tight linear relationship between the number of rearrangements simulated *r*, sampled at *r* = 600, 1200,···, ( 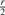 to form the ancient tetraploid *T* and 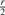 to form the modern diploid *D*) and the increase in the number of adjacencies, when the number of deletions *d* is zero.

Although we can invent examples where the number of adjacencies and the number of rearrangements are less predictive of each other, these would be encountered with negligible frequency in real data or in simulations. In the ensuing sections, therefore, we will use the number of excess adjacencies as a proxy for the amount of rearrangement.

### 2.3 The effects of fractionation on adjacencies

**Fig. 4.**
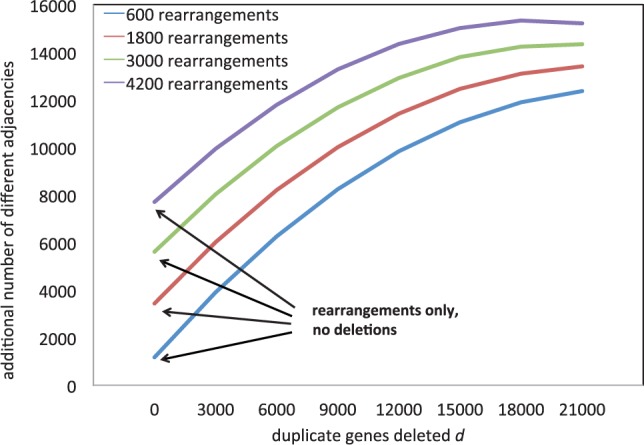
Simulated increase in apparent rearrangement in an ancient tetraploid compared to a diploid sister genome, as a function of actual rearrangements *r* and number of deleted duplicate genes *d*. Vertical axis represents increase in total number of different adjacencies in *D* and *T* combined, compared with the 24 000 in the ancestral genome. Samples at *d* = 0,3000,6000,…

We carried out extensive simulation experiments to assess how the process of fractionation impacts the apparent amount of rearrangement. The genes deleted to form the ancient tetraploid were chosen entirely at random from the entire genome, under the constraint that once a gene was deleted, its duplicate could not be deleted in a later step. The solid lines in Figure 4 show the effect of increasing amounts of fractionation on the apparent amount of rearrangement, a gross distortion.

## 3 ALGORITHM

**Fig. 5.**
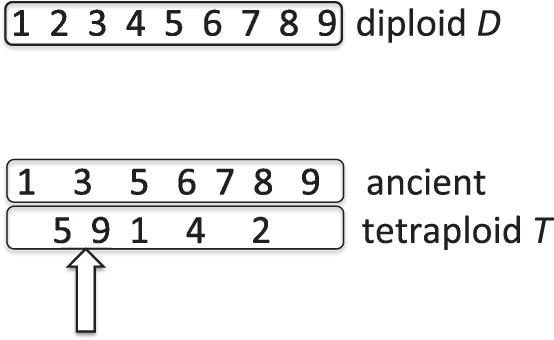
Arrow indicates positioning of third (empty) fractionation interval for genes 6,7 and 8, on the evidence of the positioning of these three genes between 5 and 9 in the diploid and on one homeologous chromosome

We developed the consolidation algorithm to assess and correct for this distortion. This focuses on detecting and accounting for two homeologous regions of single-copy genes in the ancient tetraploid which contain no genes in common (as the genes concerned are single copy) but whose combined [or *consolidated* (Langham *et al*., 2004)] gene content is exactly the same as some contiguous region in the diploid. We call maximal regions like this Class 1 regions, in contrast to Class 2 regions, where there is only one region in the tetraploid which contains only, and exactly, the same single-copy genes as a region in the ancient tetraploid, but where there is sometimes clear adjacency evidence of where the duplicate copies of these genes were deleted, as in Figure 5. Classes 1 and 2 exhaust all the single-copy genes in the ancient tetraploid. The algorithm for detecting and listing these regions runs in linear time in the number of genes. Once the regions are identified, the three components of a Class 1 region are replaced by a new, labelled, dummy gene. Similarly, for the two components of a Class 2 region, with a third copy of the dummy gene placed whenever possible according to the afore-mentioned adjacency evidence.

The two genomes, thus reduced by the replacement of single-copy genes by a lesser number of dummy genes, is then examined for excess adjacencies.

Note that the genes in this discussion are all oriented, i.e. their 5′-and 3′-ends are distinguished, and when we speak of two genes being adjacent, we mean that the 3′-end of one is adjacent to the 5′-end of the next one, if they are on the same DNA strand or that their two 5′-ends (or two 3′-ends) are adjacent if they are on complementary strands. An adjacency is thus defined by two specific gene ends and not by two genes. Nevertheless, in the ensuing discussion, we may sometimes speak of adjacent genes without introducing any ambiguity.

### 3.1 Consolidation algorithm

**Input:** Triple (*L,D,T*), where

- *L* is a set of genes.
- *D* is a diploid genome, i.e. *L* is partitioned among a number of chromosomes; the genes on a chromosome are ordered.
- *T* is an ‘ancient’ tetraploid, i.e. each gene of *L* occurs in either one or two copies; the genes are partitioned and ordered on a number of chromosomes, with no constraint on positions of two copies of the same gene.
- For each gene *g* ∈ *L*, pointers [*C*_1_(*g*),*p*_1_(*g*),*C*_2_(*g*),*p*_2_(*g*), *C*_3_(*g*),*p*_3_(*g*)] indicate its chromosomal membership and its position in that chromosome's order. The subscript ‘1’ refers to *D* and ‘2’ and ‘3’ refer to the occurrences of *g* in *T*, where *C*_3_(*g*) = *p*_3_(*g*) = ∅ if *g* occurs only once in *T*.

**Output:** Triple (*L*^′^,*D*^′^,*T*^′^), where

- *D*^′^ is a diploid genome with gene set *L*^′^, and *T*^′^ is a ‘pure’ tetraploid (all genes occur exactly twice) on *L*^′^.
- *L*^′^ = *L*_2_ ∪ *L_V_*. *L*_2_ ⊆ *L* is the set of genes with two copies in *T*, and *L_V_* is a set of ‘virtual’ genes, each one representing a triple of intervals of genes (in *L*), (*I*_1_, *I*_2_, *I*_3_), the first, *I*_1_, on a chromosome in *D*, the second and third on chromosomes in *T*, such that *I*_2_ ∩ *I*_3_ = ∅, *I*_2_ ∪ *I*_3_ = *I*_1_. The *I*_1_ from different virtual genes are disjoint, and their union is *L*\*L*_2_, the entire set of single-copy genes in *T*. Moreover, the elements of *L_V_* are jointly maximal in that the union of no two of them can be a virtual gene.

#### Detect fractionation intervals

While there are still single-copy genes in *T* that have not been placed intervals, create new element of *L_V_* as follows

- Initialize empty intervals *I*_1_, *I*_2_ and *I*_3_. Find the first single-copy (in *T*) gene in *D*, add it to *I*_1_ and *I*_2_.
- While the next gene in *D* is a single-copy (in *T*) gene on the same chromosome as the other genes in *I*_1_ and it is on the same chromosome as *I*_2_ or *I*_3_, add it to *I*_1_ and to *I*_2_ or *I*_3_. If it is not on the same chromosome as *I*_2_, and *I*_3_ is empty until the present time, the gene determines on which chromosome the interval *I*_3_ is to be located.
- While the genes in *I*_2_ (or *I*_3_) are not contiguous, remove the last gene assigned to *I*_1_ and *I*_2_ or *I*_3_.
- If *I*_3_ ≠ ∅, the virtual gene *g* representing the triple (*I*_1_, *I*_2_, *I*_3_) is added to *L_V_*, and it replaces the genes in *I*_1_ on *D*, and the genes in *I*_2_ and *I*_3_ on *T*.
  Else (if *I*_3_ = ∅),
- **place** *I*_3_
  Consider the two genes (actually gene ends) *a* and *c* adjacent on the left and right of *I*_1_ in *D* and the two genes *b* and *d* adjacent on the left and right of *I*_2_ in *T* . Locate all further copies of *a, b, c* and *d* in *T*, and insert *I*_3_, or the virtual gene representing *I*_3_ into *T* adjacent to one, or between two, of these gene ends, in such a way as to minimize the number of new adjacencies thus created. This involves trying all eight combinations or orientations for the virtual genes in its three locations
- Now the virtual gene *g* representing the triple (*I*_1_, *I*_2_, *I*_3_) can be added to *L_V_*, replacing the genes in *I*_1_ on *D*, and the genes in *I*_2_ and *I*_3_ on *T*.
- There are minor details, which we will omit, on handling ‘telomeric’, i.e. at the ends of chromosomes, genes.

Note that **place** *I*_3_ contains an optimization step. The rest of the algorithm is deterministic, and it may very well not call **place** *I*_3_ at all. Nevertheless, the consolidation algorithm may be reformulated as a combinatorial optimization problem, where the objective is to minimize the number of different adjacencies by ‘placing’ all the empty *I*_3_ intervals at the same time.

The consolidation algorithm, as presented, has quadratic worst case running time, in the number of genes, but in practice, on large genomes both real and simulated, it takes seconds or minutes. Other consolidation algorithms, with linear worst case running time, have been devised by J. Kováč (personal communication) and K. Jahn (personal communication).

The consolidation algorithm treats the fractionation intervals as identical units, one in the diploid and two in the tetraploid. In this way, it accounts not only for rearrangements which includes a whole interval in its scope but also for rearrangements which disrupt an interval, in that a fractionation involving such an interval will generally be automatically counted as two intervals, resulting in two virtual units instead of one. What the consolidation algorithm does not account for, however, are rearrangements occurring completely within one of the fractionation intervals.

To correct for this, within each fractionation regions, we first consider all the adjacencies in *I*_1_ and compare each *I*_2_ and, if necessary, *I*_3_, with *I*_1_. For the comparison, with *I*_2_, for example we delete the genes in *I*_1_ not in *I*_2_. We count only those *a*_2_ adjacencies in *I*_2_ not in the reduced *I*_1_. We add the number of these ‘rearranged’ adjacencies to the set of adjacencies produced by the consolidation algorithm.

## 4 RESULTS AND DISCUSSION

### 4.1 Applying the algorithm to simulated genomes

**Fig. 6.**
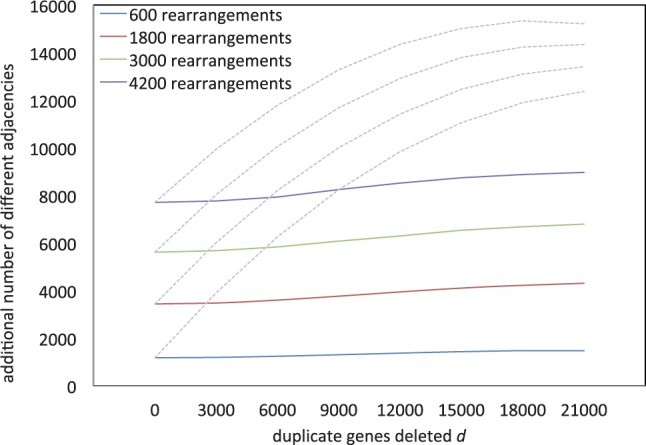
Solid line: apparent amount of rearrangements stable after application of consolidation algorithm and taking into account adjacencies within fractionation regions. Dashed line: before algorithm

As can be seen in Figure 6, for moderate rearrangement distances, and for a wide range of fractionation rates, our procedure almost completely wipes out the distortion caused by the fractionation.

### 4.2 The study of fractionation regions

The consolidation algorithm was motivated as a way of correcting estimates of genomic divergence, but an important byproduct is its systematic identification of fractionation regions. This will be the focus of the remainder of this article, including its empirical aspect. First, we will explore fractionation regions in the contexts of our simulations and in the next section, discuss how to analyze this process in the core eudicots.

### 4.3 Why can not we just ignore fractionation regions?

The consolidation algorithm adapts genomic rearrangement assessment to the case of mixed duplicate and single-copy genes, through the device of virtual genes representing fractionation regions. We may ask, what if we just delete all single-copy genes and compare the diploid sister genome with whatever duplicates remain in the ancient tetraploid? This question is motivated by the original approach (since abandoned) to the ‘guided genome halving’ problem (Zheng *et al*., 2007).

**Fig. 7.**
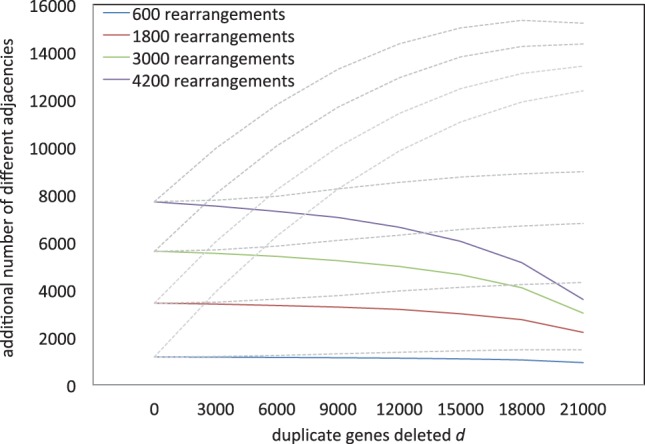
Solid lines: change in apparent rearrangement in an ancient tetraploid compared with a diploid sister genome, as a function of actual rearrangements and number of deleted duplicate genes, when single-copy genes are ignored. Dashed lines: including single-copy genes, before and after application of consolidation algorithm

The results, shown in Figure 7, show that this alternative approach is not a satisfactory way of handling single-copy genes, considering its substantial underestimate of the amount of rearrangement, which is especially severe when the fractionation process is far advanced.

### 4.4 Rearrangements and fractionation region size

**Fig. 8.**
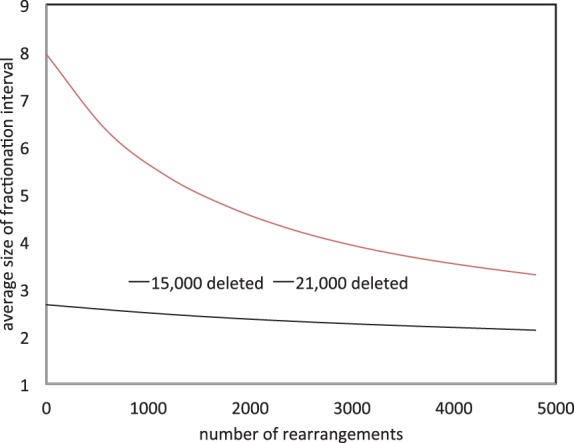
Fractionation region size as a function of number of rearrangements

When only a few of the homeologous gene pairs have been reduced to single copy, fractionation regions will tend to be small and dispersed across the genome. Most rearrangements will not change the contents of any fractionation region. When most of the gene pairs have been reduced to single copy, fractionation regions will tend to be longer, especially if there have been few rearrangements. Then each new rearrangement is likely to disrupt a fractionation region, usually breaking it into two. These tendencies can be seen in Figure 8, where the fractionation regions for a genome with 15 000 out of 24 000 gene pairs reduced to single copies, are little affected by rearrangements. Where 21 000 of the pairs are thus reduced, the size of the fractionation regions is much more sensitive to rearrangements.

### 4.5 Fractionation in the poplar genome

**Fig. 9.**
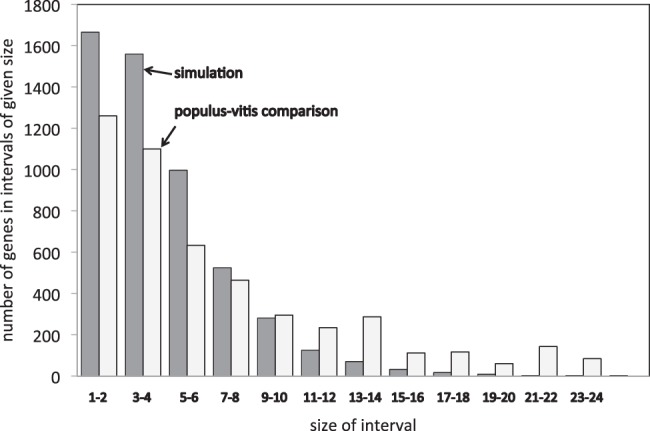
Size of *Populus–Vitis* fractionation regions compared with simulations matched for rearrangement and deletion rates. In *Populus–Vitis*, the single-copy genes are concentrated in fewer, larger regions

In applying our method to the genome of poplar (*Populus trichocarpa*) (Tuskan *et al*., 2006), an ancient tetraploid, compared with diploid sister genomes, grapevine (*Vitis vinifera*) (Jaillon *et al*., 2007) and cacao (*Theobroma cacao*)^1^ (Argout *et al*., 2011), we discovered that the large majority, 70–90%, of the apparent rearrangements are actually attributable to fractionation. We also carried out matching simulations, with the same number of duplicate and single-copy genes, produced by random paralog deletion and a number of random rearrangements, mostly inversions, to produce the same number of excess adjacencies. Examining the consolidated regions detected by our algorithm, there are a number of regions much longer than those in the simulations (Fig. 9), suggesting a non-independence of deletion events affecting neighboring genes and clear tendency for genes to be deleted in one of the two homeologs, as would be predicted by the recent theory of subgenome dominance (Schnable *et al*., 2011): ‘Genes are disproportionately lost from one parental subgenome, the subgenome that is less expressed in the polyploid’.

### 4.6 Effect of deletion event run size

Motivated by the discrepancy between the poplar genome fractionation region sizes and the results of the simulations, we ran new simulations where instead of choosing duplicate genes to delete one at a time at random, we deleted one or more contiguous genes at a time, starting at a random position in the genome, according to a geometric distribution with mean *μ*. To model the quantitative bias in the fractionation process, successive genes were deleted from the same homeolog with ‘continuation’ probability *p* ≥ ½ and from the opposing homeolog with probability 1 – *p*.

To infer the best values of the parameters *μ*,*p* and the *r* number of rearrangements to account for the poplar–*Vitis* and poplar–cacao comparison, we simulated the evolutionary process with a fixed number of genes in the diploid and tetraploid and a fixed number of single-copy genes in the tetraploid. We searched in a three-dimensional grid where *μ ∈* [1, 2],*p ∈* [0.5, 1.0] and *r* takes on a wide range of values depending on *μ*, for the values of the parameters that could produce the same number of adjacencies before and after the consolidation algorithm and the same number of fractionation regions as the real data. The results for

- The real genomes,
- A model where one gene is deleted at a time (*μ* = 1,*p* = 1),
- The best model with geometrically distributed deletion lengths,
- A model with *μ* = 2,

are compared in Table 1.

**Table 1.** Inference (in boldface) of deletion event size parameter *μ*, continuation parameter *p* and number of rearrangements *r*, necessary to model poplar–*Vitis* and poplar–cacao comparisons. Adj_1_: adjacencies before consolidation, Adj_2_: adjacencies after consolidation, *f*: number of fractionation regions, 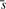: average size of fractionation regions

*μ*	*r*	*p*	Adj_1_	Adj_2_	*f*	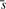
*Populus:* 18 309 genes, 5287 single-copy, *Vitis:*11 798 genes						
real genome data:			16 877	10 988	2356	2.24

1	680	1	16 866	10,970	3005	1.76
**1.7**	**1040**	**0.5**	**16 900**	**10,957**	**2355**	**2.23**
2	1120	0.5	16 888	10,941	2184	2.42

*Populus:* 18 221 genes, 5557 single-copy, *Cacao:* 11 889 genes						
real genome data:			16 357	10 279	2297	2.42
1	320	1	16 618	10 074	3014	1.84
**1.8**	**800**	**0.7**	**16 433**	**10 345**	**2297**	**2.42**
2	840	0.7	16 371	10 330	2195	2.53

The results from the two diploids are similar with respect to *μ*, namely 1.7 and 1.8, but the *p* are rather different: 0.5 for *Vitis* and 0.7 for cacao. The most important fact is that in taking into account the sizes of the fractionation regions in the simulations requires us to increase the number of rearrangements substantially, by 50% in the case of *Vitis* and by over 100% for the cacao comparison.

**Fig. 10.**
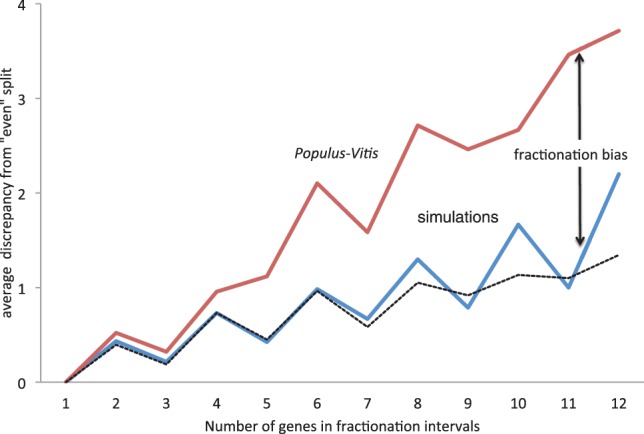
Discrepancy in two *Populus* intervals in the number of genes, compared with random simulations. In *Populus* regions, the single-copy genes are concentrated in one of the two homeologous intervals. Jagged nature of graphs due not to statistical fluctuation but to measurement of discrepancy from an ‘even’ split, which is necessarily calculated slightly differently for even and odd totals of genes in the fractionation intervals. Black dashed line represents simulations with deletion event run size geometrically distributed with mean 1.7

As we built no provision into the simulations for fractionation to affect the two homeologs differently there is no reason to expect the kind of fractionation bias we observed in poplar to show up in the simulations. Indeed, Figure 10 shows that simulations with or without deletion event run size variability do not differ. This is further confirmation of the reality of the observed effect that it is not an artifact of duplicate deletion regime nor of the consolidation algorithm.

### 4.7 Single-copy region syntenies with cacao and *Vitis*

Since the cacao and *Vitis* genomes have a long history of independent rearrangements, we can expect that fractionation regions induced by the consolidation algorithm by one of them in poplar will generally differ from the regions determined by the other. Nevertheless, we found many regions in the *Vitis*–poplar comparison that overlapped substantially in poplar with regions in the cacao–poplar comparison. In particular, we found eight regions that contained more than 15 genes in both comparisons; six of these pairs of comparisons had more than 15 genes in common; of most interest is that the common genes were 100% on only one homeolog in seven of the eight cases. This strongly suggests that there is a functional reason for these large sets of contiguous genes to be reduced to single-copy and for these copies to be retained on a single homeolog.

### 4.8 Subgenome dominance

Although we have detected and measured substantial bias in the fractionation of ancient core eudicot tetraploids, without a gene-by-gene reconstruction of the ancestral chromosomes, we cannot as yet be sure that the bias is systematically in favor of one or other of the diploids contributing to the original polyploid. This dominance of one of the subgenomes has been demonstrated in the case of maize (Schnable *et al*., 2011), where it was also correlated to gene expression, patterns which persisted despite a number of genome rearrangement events disrupting the original synteny relations among genes.

## 5 DISCUSSION

An analytically advantageous feature of our analysis is that it partitions the rearrangements that have affected a tetraploid into those that have operated within a fractionation interval and those that have left these intervals intact, either because the intervals are outside the scope of the rearrangement or the interval is affected as a whole, without any effect internally. Of course, in the the history of the tetraploid there will have been some rearrangements that have involved elements both within and outside fractionation intervals, but these are rendered irrelevant and invisible to our analysis because their effect is simply to fragment the intervals into two or more new intervals and hence perpetuate the within-outside partitioning of the rearrangements.

One of the problems remaining with this work is the large number of genes that do not appear in any synteny block in the diploidtetraploid analysis of pairs of core eudicot genomes. This despite reducing the minimum block length as low as 2 in SynMap. Some of this is undoubtedly due to genes absent from one of the genomes, but much of it will be due to movement of genes out of their erstwhile homeologous contexts, by various processes (Wicker *et al*., 2010; Woodhouse *et al*., 2011).

In our simulations of geometric deletion events, we ‘marked’ some duplicate genes for deletion before carrying out the rearrangement step, and only actually deleted them afterward. This was for purposes of comparability of the results with the previous simulations where deletions followed rearrangements. Our goal in this marking was to allow a degree of switching between homeologous chromosomes during the deletion event, and no connection was assumed between two different events. However, this computational device could also be used to model subgenome dominance, by using two different *p*-values one for the dominant subgenome and a smaller value for the other subgenome. Marking becomes analogous to (lack of) methylation or to other epigenetic regulatory mechanism that can persist despite extensive genome rearrangement.

It will be of interest to explore the extensions of our treatment of fractionation to genomes descending from more complex polyploidization events than WGD. The plant genomes studied here, as well as all the other core eudicots, descend from an ancestral hexaploidization event (Jaillon *et al*., 2007). Extensive fractionation has left only a hundred or so triplicates intact and about a thousand duplicates, in *Vitis* and in cacao (Zheng *et al*., 2012). Many core eudicot angiosperm genomes have been sequenced but as of yet no sequence of a dicot that diverged before the hexaploidization event has been published, so that extensions of our procedure could be tested.

## Footnotes

^1^Data were drawn from CoGe (Lyons and Freeling, 2008; Lyons *et al*., 2008) and processed using the SynMap procedure, with a 2:1 syntenic depth setting to account for the ancient tetraploidy of poplar, and minimum syntenic block length 2 (to avoid the excessive noise with minimum size 1 but capturing more orthologies than with higher minimum sizes).
